# P23 A survey of physician practice, knowledge and attitudes towards penicillin allergy labels and penicillin desensitization in the intensive care unit

**DOI:** 10.1093/jacamr/dlag102.029

**Published:** 2026-06-26

**Authors:** John E A Taylor, Neil Powell, Andrew F Whyte, Rob Jackson, Jennie Stephens, Jonathan Sandoe

**Affiliations:** Southwest Peninsula Deanery, UK; Royal Cornwall Hospitals NHS Trust, Truro, UK; University Hospitals Plymouth NHS Trust, Plymouth, UK; University Hospitals Plymouth NHS Trust, Plymouth, UK; Royal Cornwall Hospitals NHS Trust, Truro, UK; University of Leeds, Leeds, UK; Leeds Teaching Hospitals NHS Trust, Leeds, UK

## Abstract

**Background:**

Penicillin allergy labels are reported in approximately 10% of patients, yet over 90% of these labels are inaccurate when formally tested. This mislabelling has important implications for antimicrobial stewardship, as it leads to avoidance of first-line β-lactam antibiotics, resulting in the unnecessary use of broad-spectrum alternatives, increased risk of resistant infections, and higher healthcare costs. In intensive care settings, these issues are magnified due to the complexity of infections and the urgency of empirical antibiotic therapy. Penicillin desensitization is a recognized but underused strategy that allows safe administration of β-lactams in potentially allergic patients.

**Objectives:**

To evaluate the knowledge, clinical practices, and attitudes of intensive care physicians towards penicillin allergy labels and the use of penicillin desensitization within the ICU. Identifying perceived barriers to desensitization, assessing access to specialist allergy services, and exploring the frequency of inappropriate penicillin avoidance. The findings were intended to inform the potential development of penicillin desensitization protocols for use in critically ill patients.

**Methods:**

An anonymous online survey was distributed to U.K. ICU clinicians via the Faculty of Intensive Care Medicine eNewsletter between February 2020 and August 2021. The data was analysed using frequency and percentage distributions, and free-text responses were coded thematically to explore qualitative insights into barriers and perceived risks.

**Results:**

A total of 118 responses representing 33 U.K. hospitals were received; 99% of respondents were doctors and 57% were consultants. Respondents estimated that between 5% and 20% of ICU patients report a penicillin allergy (mean estimate 13%), consistent with published epidemiological data. The majority believed that many labelled patients could safely tolerate penicillin, with 52% estimating that 51–75% would tolerate treatment and 8% estimating that 76–100% would tolerate penicillin therapy. In current practice, 85% of clinicians reported that they ‘sometimes’ or ‘often’ administer penicillin to patients with an allergy label when the history suggests a low likelihood of true allergy. Despite this, referral to allergy or immunology services was uncommon. Access to allergy or immunology services was limited, with only 31% of respondents reporting on-site availability, although microbiology support for antimicrobial decision-making was widely available. Experience with penicillin desensitization was very limited; 90% of respondents had never undertaken the procedure in the ICU. The most reported barriers were lack of local allergy or immunology expertise (51%), lack of knowledge or familiarity with the procedure (39%), concerns regarding safety in critically ill patients (26%) and perceived workload for nursing staff (24%).

**Conclusions:**

The results highlight a significant gap in awareness and capability regarding penicillin desensitization amongst ICU clinicians in the U.K. Despite widespread recognition of the importance of accurate allergy assessment, desensitization remains rare due to limited local expertise, lack of clear protocols, and misconceptions regarding safety. Studies have shown that structured allergy testing and desensitization programmes can safely broaden antibiotic options, reduce MDR infections, and shorten hospital stays. Improved collaboration between ICU and allergy services, standardized pathways, and educational initiatives could enhance patient outcomes and stewardship efforts.

